# Supplementary figures and images for: Lack of parent-of-origin effects in Nasonia jewel wasp: A replication and extension study
Source: PLoS One. 2021 Jun 10;16(6):e0252457. doi: 10.1371/journal.pone.0252457 (PMC8191985; doi:10.1371/journal.pone.0252457)

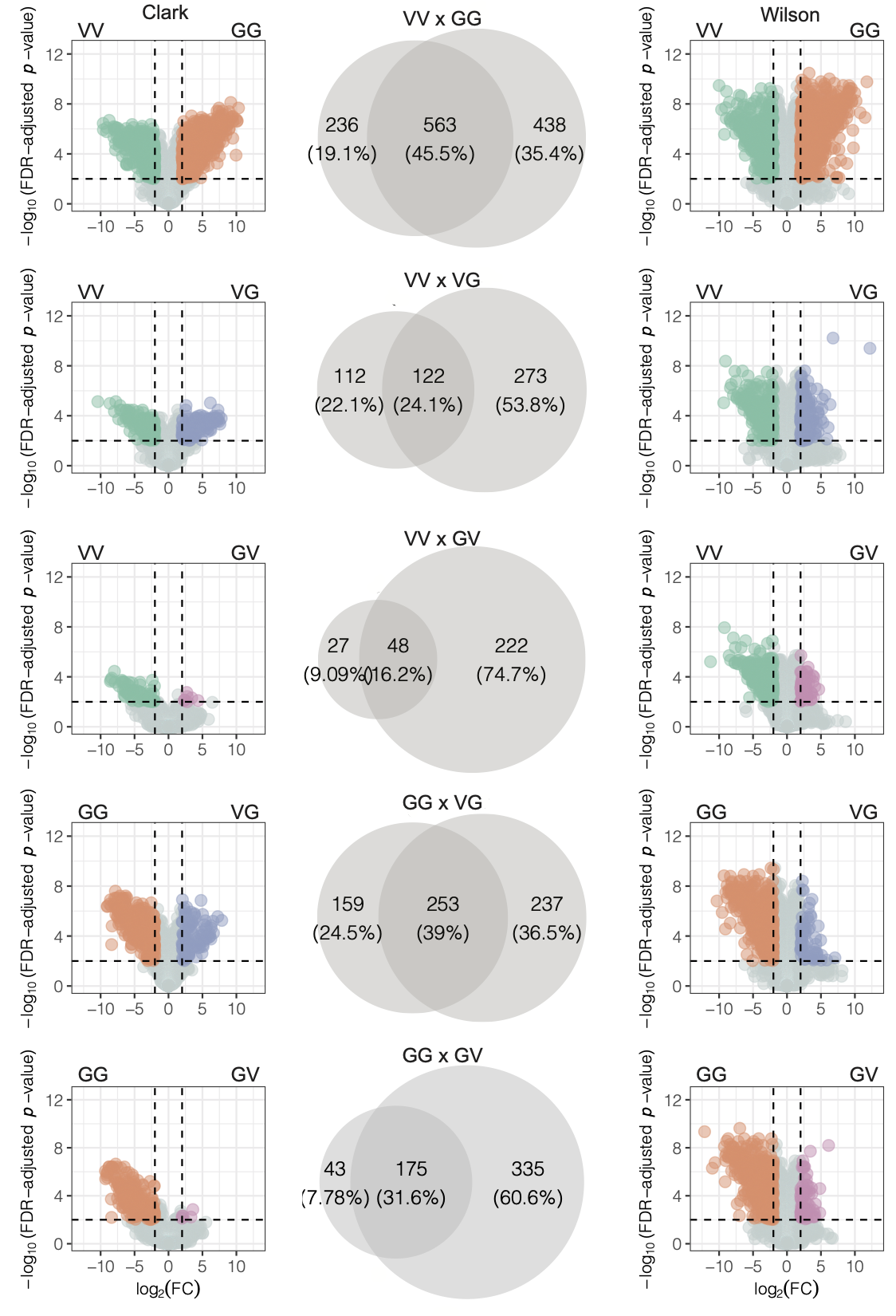

Supplement: S1 Fig — Volcano plots of DEGs detected between the different comparisons involving N. vitripennis, N. giraulti, and the two reciprocal F1 hybrids in the R16A Clark (left side) and Wilson (right side) datasets. Venn diagrams of the overlap of significant DEGs in each comparison is shown. (TIFF) [file pone.0252457.s001.tiff]

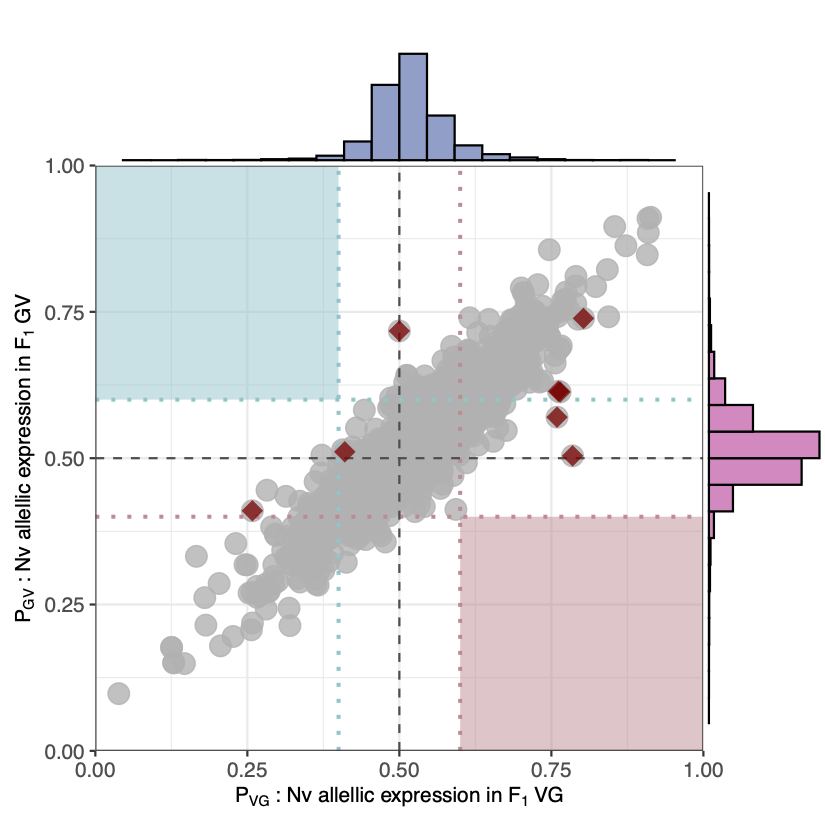

Supplement: S2 Fig — Scatterplots of the expression of the N. vitripennis alleles in the two reciprocal hybrids, VG (x-axis) and GV (y-axis). Analysis was limited to 5,759 genes with at least 2 informative SNPs in the reciprocal hybrids in the combined R16A Clark and Wilson dataset. Genes exhibiting a significant difference in allelic bias between the hybrids (Fisher’s exact test, FDR-adj. p<0.05) are highlighted in red. Paternally imprinted genes are expected to appear in the upper left corner (light blue box), and maternally imprinted genes in the lower right corner (light pink box). Histograms of the N. vitripennis allele expression are shown for VG (blue) and GV (pink). (TIFF) [file pone.0252457.s002.tiff]
